# Retraction Note: Effects of innate immune receptor stimulation on extracellular α-synuclein uptake and degradation by brain resident cells

**DOI:** 10.1038/s12276-026-01789-x

**Published:** 2026-06-29

**Authors:** Changyoun Kim, Somin Kwon, Michiyo Iba, Brian Spencer, Edward Rockenstein, Michael Mante, Anthony Adame, Soo Jean Shin, Jerel A. Fields, Robert A. Rissman, Seung-Jae Lee, Eliezer Masliah

**Affiliations:** 1 Molecular Neuropathology Section, Laboratory of Neurogenetics, National Institute on Aging, National Institutes of Health, Bethesda, MD 20892 USA; 2 Department of Neurosciences, School of Medicine, University of California, La Jolla, San Diego, CA 92093 USA; 3 Department of Biomedical Sciences, Neuroscience Research Institute, and Department of Medicine, Seoul National University College of Medicine, Seoul, 03080 Korea; 4 Department of Psychiatry, School of Medicine, University of California, La Jolla, San Diego, CA 92093 USA; 5 Division of Neuroscience, National Institute on Aging, National Institutes of Health, Bethesda, MD 20892 USA

Retraction Note to: *Experimental & Molecular Medicine* 10.1038/s12276-021-00562-6, published online 16 February 2021

The Editor-in-Chief has retracted this article. After publication, concerns were raised regarding areas of high similarity in the Fig. 3b Neocortex (High) α-syn-tg LV-con image.

The authors have provided the full-size image files used in Fig. 3b for validation. However, further checks by the publisher found additional cases of high similarity areas in these images. The Editor-in-Chief therefore no longer has confidence in the presented data.

Soo Jean Shin and Seung-Jae Lee agree with this retraction. Eliezer Masliah does not agree with this retraction. Edward Rockenstein is deceased. The other authors have not responded to any correspondence from the editor or publisher about this retraction.

